# Epidemic of lower extremity peripheral arterial disease in China: current trends and future prediction

**DOI:** 10.3389/fcvm.2025.1571146

**Published:** 2025-06-11

**Authors:** Jianxing Yu, Yuhan Zhang, Qingqing Zhang, Jinyi Wang, Aiqin Gu, Jing Wei, Chuanmeng Zhang

**Affiliations:** ^1^ School of Medicine, Linyi University, Linyi, Shandong, China; ^2^ Clinical Medicine Eight Year Program, Southern Medical University, Guangzhou, Guangdong, China; ^3^ Pan-Vascular Management Center, The Affiliated Taizhou People’s Hospital of Nanjing Medical University, Taizhou, Jiangsu, China; ^4^ Clinical Research Center, Taizhou School of Clinical Medicine, The Affiliated Taizhou People’s Hospital of Nanjing Medical University, Nanjing Medical University, Taizhou, Jiangsu, China; ^5^ Department of Obstetrics and Gynecology, The Affiliated Taizhou People’s Hospital of Nanjing Medical University, Taizhou, Jiangsu, China

**Keywords:** lower extremity peripheral arterial disease, disease burden, prediction, APC analysis, BAPC analysis

## Abstract

**Background:**

Lower extremity peripheral arterial disease (PAD) reflects the overall condition of the cardiovascular system. Due to its often asymptomatic nature, PAD is frequently overlooked. We aimed to estimate the disease burden of PAD in China over the past 30 years and to project future trends over the next 25 years.

**Methods:**

The incidence and disability-adjusted life years (DALYs) of PAD was extracted from the Global Burden of Disease (GBD) database and subsequently described. Joinpoint regression was used to assess trends from 1990 to 2021, and an age-period-cohort model was constructed to examine the influence of period and cohort effects on incidence and DALYs of PAD. A Bayesian APC model was also applied to forecast trends through 2046.

**Results:**

In 2021, the annual number of new PAD cases in China was 2.45 (95% UI: 2.11–2.85) million, of which 1.74 (1.50–2.03) million were female and 0.71 (0.61–0.83) million were male. The number of new cases in 2021 was obviously higher than that in 1990 among females and males. The age-standardized incidence rate (ASIR) exhibited an increasing trend among males, while a decreasing trend was observed among females. Incidence number rose across all age groups, but rates declined in females. Period effects were identified as high-risk factors for PAD incidence and in both sexes, whereas the cohort effects appeared protective. The number of new cases is projected to rise from 2.45 million in 2021 to 4.04 million by 2046, while the ASIR remains stable. Trends in DALYs showed similar patterns.

**Conclusion:**

The burden of PAD in China has increased markedly from 1990 to 2021 and is expected to continue rising over the next 25 years. Efforts to reduce modifiable risk factors-such as smoking and metabolic diseases-and to enhance PAD prevention and management, including the establishment of Pan-Vascular Management Center, are urgently needed.

## 1 Introduction

In China, the aging population and increasing prevalence of vascular risk factors‒including hypertension, diabetes, and dyslipidemia‒have drawn growing attention to vascular diseases (1). Lower extremity peripheral arterial disease (PAD) is a progressive atherosclerotic condition characterized by arterial stenosis or occlusion of the lower limbs (2–4). Clinical features include intermittent claudication, ischemic pain, functional impairment, and increased risk of cardiovascular events and mortality, all of which contribute to reduced quality of life (2, 5). Globally, PAD represents a mounting public health challenge (6), with an estimated 236 million individuals affected (7, 8). Over the past three decades, the disease burden has nearly doubled (9). However, PAD is frequently underdiagnosed and undertreated due to its often asymptomatic presentation (6).

In recent years, the establishment of 300 Pan-Vascular Management Center (PVMC) in China has signaled a shift toward more integrated vascular care, which includes early detection and management of PAD (10, 11). Coronary artery disease, cerebrovascular disease, and PAD all fall under the category of pan-vascular diseases, characterized by a similar type of atherosclerotic pathology affecting different vascular beds. The rupture of atherosclerotic plaques can lead to varied clinical manifestations depending on the organs involved, including unstable angina, myocardial infarction, ischemic stroke, severe limb ischemia, cardiovascular mortality (1). Furthermore, PAD is considered one of the indicators of the severity of systemic vascular disease (1). Thus, understanding the current epidemiological status and future trends of PAD in China is essential for the management of pan-vascular disease, allocation of healthcare resources and the formulation of health policies. In this study, we analyzed the epidemiological status of the incidence and disability-adjusted life years (DALYs) of PAD in China from 1990 to 2021 based on the Global Burden of Disease (GBD) database and predicted future trends using the Bayesian age-period-cohort (BAPC) model.

## 2 Methods

### 2.1 Data source

The GBD 2021 study provides data on the incidence, prevalence, years lived with disability, DALYs, and healthy life expectancy for 371 diseases and injuries across 204 countries and regions, as well as 811 subnational entities (12). This analysis employs the most recent epidemiological data and refined standardization methods, including the Disease Model-Bayesian Meta-regression, the Cause of Death Ensemble Model, and Spatiotemporal Gaussian Process Regression (13). In this survey, we utilized the GBD 2021 database (https://vizhub.healthdata.org/gbd-results/) to extract the disease burden of PAD (ICD-10 codes: I70.2 and I73.9) in China from 1990 to 2021, stratified by age group and gender. Uncertainty intervals (UIs) were used to indicate variability in the estimates.

Chinese GBD estimates were derived from a range of sources, including censuses, surveys, the Disease Surveillance Point system, the China Cancer Registry, the Maternal and Child Surveillance System, and the Chinese Center for Disease Control and Prevention cause-of-death reporting system. Additional sources included data from the Hong Kong Special Administration Region (SAR), Macao SAR, and peer-reviewed publications (13, 14). Data on nonfatal outcomes were predominantly collected from national surveys, hospital inpatient data, the China Cancer Registry, and the cause-of-death reporting system of the Chinese Center for Disease Control and Prevention, in addition to published papers or reports (13, 14). The population representativeness and reliability of the data have been officially recognized, and this data has been utilized in several studies published in top-tier journals (13, 15, 16).

### 2.2 Joinpoint regression analysis

The Joinpoint regression model was employed using Joinpoint software (version 5.2.0). Time was the independent variable, while number and age-standardized rate (ASR) of incidence and DALYs of PAD served as dependent variables (17). It establishes a segmented regression based on the temporal characteristics of disease burden, fitting, and optimizing trends for each interval to assess the changes in disease characteristics over a specified timeframe (18). The annual percentage change (APC) for each interval, average APC (AAPC) across the entire range, and corresponding 95% confidence intervals (CIs) were estimated to assess changes in PAD disease burden. An APC or AAPC greater than 0 with a 95% CI greater than 0 indicated an upward trend in the disease burden; conversely, an APC or AAPC less than 0 with a 95% CI less than 0 indicated a downward trend. Otherwise, no significant changes were observed.

### 2.3 Age-period-cohort (APC) analysis

An APC model was constructed to investigate the influence of period and cohort effects on the incidence and DALYs rates of PAD. This statistical model is widely employed in epidemiological research and its specific formulation is as follows (19, 20):

$$Y=\log(M)=b+\mathrm{mAg}e_{i}+\mathrm{nPerio}d_{j}+\mathrm{pCohor}t_{k}+e$$

where *Y* denotes the outcome variable; m, n, and p represent the coefficients for age, period, and cohort effects within the APC model; b indicates the model intercept; and e denotes the residual term of the APC model.

### 2.4 Bayesian age-period-cohort (BAPC) analysis

The BAPC model incorporates prior distributions with sample data to estimate posterior distributions and infer unknown parameters (21). Existing research has demonstrated that the BAPC model outperforms other predictive methods in terms of coverage and accuracy (22, 23). In this study, we employed a BAPC model incorporating integrated nested Laplace approximation (INLA) to predict future trends in disease burden of PAD. Thus, the open-source software R (version 4.2.1), along with the R packages BAPC and INLA, was utilized to forecast the trends in China from 2022 to 2046.

## 3 Results

### 3.1 Overview and trend analysis of the disease burden of PAD in China

**Figure 1:**
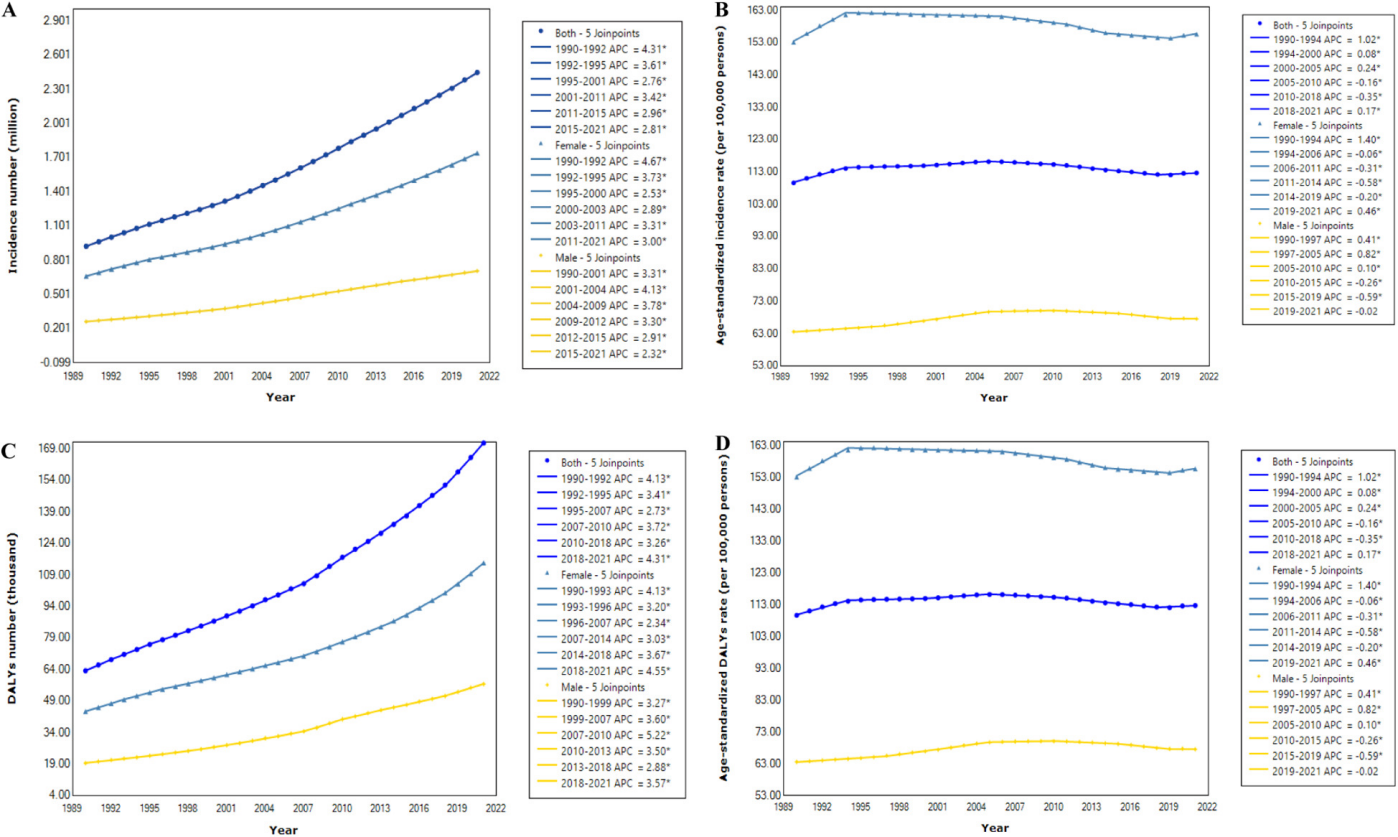
Joinpoint regression analysis in incidence number **(A)**, age-standardized incidence rate **(B)**, DALYs number **(C)**, and age-standardized DALYs rate **(D)** of PAD in China from 1990 to 2021. An asterisk indicates that the annual percent change is statistically significantly different from zero at the α = 0.05 level.

Table 1 presents a description and trend analysis of the incidence and DALYs associated with PAD in China from 1990 to 2021. In 2021, the annual number of new PAD cases reached 2.45 (2.11–2.85) million, including 1.74 (1.50–2.03) million females and 0.71 (0.61–0.83) million males. This number was significantly higher than that in 1990, which recorded 0.92 (0.80–1.09) million total cases-0.66 (0.57–0.77) million among females and 0.26 (0.22–0.31) million among males. Accordingly, from 1990 to 2021, the annual incidence number exhibited a consistent upward trend (AAPC = 3.17, *P* < 0.05), categorized into six phases: 1990–1992 (APC = 4.31, *P* < 0.05), 1992–1995 (APC = 3.61, *P* < 0.05), 1995–2001 (APC = 2.76, *P* < 0.05), 2001–2011 (APC = 3.42, *P* < 0.05), 2011–2015 (APC = 2.96, *P* < 0.05), and 2015–2021 (APC = 2.81, *P* < 0.05) (Figure 1A, Table 1). Notably, in both 1990 and 2021, PAD incidence among females was more than twice that among males. However, the growth rate for males (AAPC = 3.38, *P* < 0.05), slightly exceeded that for females (AAPC = 3.09, *P* < 0.05) (Figure 1A, Table 1). In contrast, the age-standardized incidence rate (ASIR) per 100, 000 population in 2021 [112.66 (97.75–130.73)] remained similar to that in 1990 [109.57 (94.84–127.13)], with no significant difference (AAPC = −0.00, *P* > 0.05, Figure 1B, Table 1). Interestingly, the ASIR for females showed a declining trend (AAPC = −0.10, *P* < 0.05, Figure 1B, Table 1), with four periods of decline (1994–2006, 2006–2011, 2011–2014, and 2014–2019 and two periods of increase (1990–1994 and 2019–2021). Conversely, the ASIR for males increased (AAPC = 0.26, *P* < 0.05, Figure 1B, Table 1), with three periods of growth: 1990–1997, 1997–2005, and 2005–2010 and three periods of decline (2010–2015, 2015–2019, and 2019–2021).

**Table 1:** An overview and trend analysis of the number (incidence: million; DALYs: thousand) and age-standardized rate (per 100, 000 persons) of PAD disease burden stratified by gender from1990 to 2021.

Disease burden	Number			ASR		
	1990	2021	AAPC	1990	2021	AAPC
Incidence						
Both	0.92 (0.80–1.09)	2.45 (2.11–2.85)	3.17 (3.14–3.21)*	109.57 (94.84–127.13)	112.66 (97.75–130.73)	−0.00 (−0.06–0.06)
Female	0.66 (0.57–0.77)	1.74 (1.50–2.03)	3.09 (3.05–3.13)*	153.09 (132.96–177.64)	155.62 (134.88–180.26)	−0.10 (−0.17–0.04)*
Male	0.26 (0.22–0.31)	0.71 (0.61–0.83)	3.38 (3.30–3.46)*	63.54 (55.18–73.76)	67.49 (58.71–78.53)	0.26 (0.17–0.35)*
DALYs						
Both	63.35 (35.29–112.73)	171.76 (99.16–301.53)	3.12 (3.06–3.18)*	8.99 (5.08–15.96)	8.36 (4.87–14.33)	−0.37 (−0.43–0.30)*
Female	43.97 (23.17–81.28)	114.75 (60.71–215.46)	2.87 (2.78–2.97)*	11.48 (6.17–21.18)	10.35 (5.52–19.06)	−0.57 (−0.65–0.48)*
Male	19.37 (12.09–31.23)	57.01 (37.84–86.96)	3.64 (3.57–3.70)*	5.08 (3.65–9.42)	6.06 (4.14–9.10)	0.22 (0.11–0.33)*

PAD, lower extremity peripheral arterial disease; ASR, age-standardized rate; DALYs, disability-adjusted life years.

* *P* < 0.05.

In 2021, the age-standardized DALYs rate of PAD reached 8.36 (4.87–14.33) per 100,000 individuals, with an estimated 171.76 (99.16–301.53) thousand DALYs. Compared to 1990, the trends in the number and age-standardized rate (ASR) of DALYs exhibited similarities to the incidence (Figures 1C,D, Table 1). The gender proportions in these indicators were also similar to those of incidence. Detailed information is provided in Table 1.

### 3.2 Description and trend analysis of the disease burden of PAD across by age group

We further analyzed the incidence and temporal trends across age groups for both sexes. The results indicate that the incidence increases rapidly with advancing age. Among females, the number of new cases significantly increased across all age groups, particularly among older adults, although the incidence rate showed a decreasing trend. Among males, both the number of cases and the incidence rate increased significantly across all age groups (Table 2). Additionally, we conducted descriptive and trend analyses of the DALYs associated with PAD across various age groups. The results were similar to those concerning the incidence. Detailed in Supplementary Table S1.

**Table 2:** The sex-age-specifc incidence number (thousand) and rate (per 100,000 persons) of PAD in 2021 and their percentage changes from 1990 to 2021.

Categories	Female		Male	
	2021	AAPC, 95% CI	2021	AAPC, 95% CI
Number				
40–44 years	60.26 (47.61–75.15)	1.31 (0.68–1.95)*	27.15 (21.77–33.73)	1.55 (0.95–2.15)*
45–49 years	131.90 (86.29–183.92)	2.78 (2.33–3.24)*	52.79 (34.67–72.17)	2.95 (2.56–3.34)*
50–54 years	208.95 (156.91–269.23)	3.46 (3.13–3.80)*	84.44 (62.76–108.83)	3.49 (3.19–3.79)*
55–59 years	250.23 (160.65–361.93)	3.17 (2.80–3.54)*	104.20 (68.65–148.68)	3.15 (2.78–3.53)*
60–64 years	216.40 (155.67–293.70)	3.03 (2.67–3.39)*	92.69 (69.29–126.60)	3.10 (2.80–3.40)*
65–69 years	299.32 (202.87–420.14)	3.01 (2.65–3.38)*	123.15 (87.26–171.20)	3.34 (3.13–3.55)*
70–74 years	240.06 (176.03–317.92)	2.73 (2.53–2.93)*	98.00 (72.54–128.30)	3.46 (3.27–3.66)*
75–79 years	161.75 (107.31–230.85)	3.11 (3.03–3.18)*	64.25 (43.18–88.77)	4.16 (3.94–4.39)*
80–84 years	100.70 (68.17–141.48)	4.06 (3.99–4.14)*	38.36 (27.21–52.34)	5.17 (5.06–5.27)*
85 + years	71.41 (47.96–97.97)	5.85 (5.74–5.96)*	21.37 (14.17–29.05)	6.58 (6.400–6.75)*
Rate				
40–44 years	135.08 (106.74–168.47)	−0.10 (−0.22–0.03)	57.86 (46.38–71.89)	0.29 (0.22–0.36)*
45–49 years	243.10 (159.03–338.97)	−0.11 (−0.23–0.02)	94.16 (61.85–128.73)	0.30 (0.22–0.38)*
50–54 years	349.90 (262.75–450.83)	−0.09 (−0.18–0.00)	138.11 (102.64–178.00)	0.28 (0.19–0.37)*
55–59 years	454.47 (291.77–657.36)	−0.08 (−0.14–0.02)*	189.85 (125.08–270.89)	0.25 (0.17–0.34)*
60–64 years	594.83 (427.89–807.32)	−0.09 (−0.15–0.04)*	253.07 (189.19–345.66)	0.25 (0.16–0.34)*
65–69 years	768.21 (520.66–1078.29)	−0.11 (−0.17–0.06)*	326.31 (231.20–453.64)	0.27 (0.16–0.37)*
70–74 years	874.86 (641.52–1158.59)	−0.13 (−0.18–0.07)*	379.01 (280.54–496.20)	0.26 (0.15–0.37)*
75–79 years	923.43 (612.62–1317.87)	−0.13 (−0.18–0.08)*	411.77 (276.76–568.95)	0.24 (0.12–0.35)*
80–84 years	905.81 (613.24–1272.70)	−0.13 (−0.18–0.08)*	442.20 (313.68–603.38)	0.23 (0.12–0.33)*
85 + years	822.06 (552.10–1127.90)	−0.12 (−0.17–0.08)*	484.54 (321.22–658.69)	0.25 (0.17–0.32)*

*P < 0.05.

### 3.3 Period and cohort relative risks of PAD disease burden

**Figure 2:**
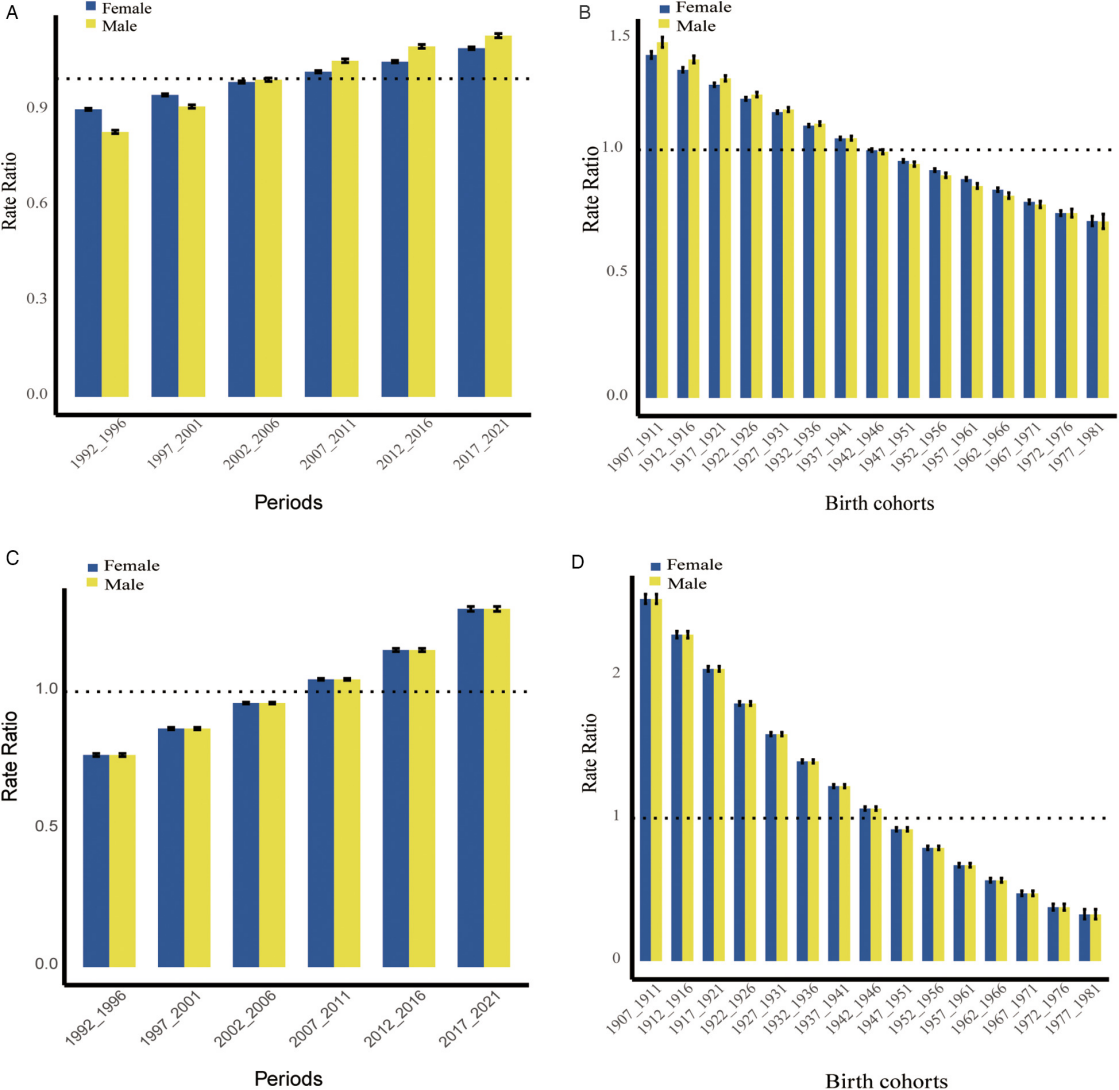
Relative risks (RRs) of PAD incidence (**(A)**: period, **(B)**: cohort)and DALYs (**(C)**: period, **(D)**: cohort) rates by sex in China.

We applied the APC model to assess the effects of age, period, and cohort on disease rate outcomes, including incidence and DALYs rates (Figure 2). Period relative risk (RR) showed a steadily increasing trend for both females (Figure 2A) and males (Figure 2C), with statistically significant differences. In contrast, cohort RR exhibited a significant decline in both sexes (Figures 2B,D).

### 3.4 Prediction of PAD disease burden in China over 25 years

**Figure 3:**
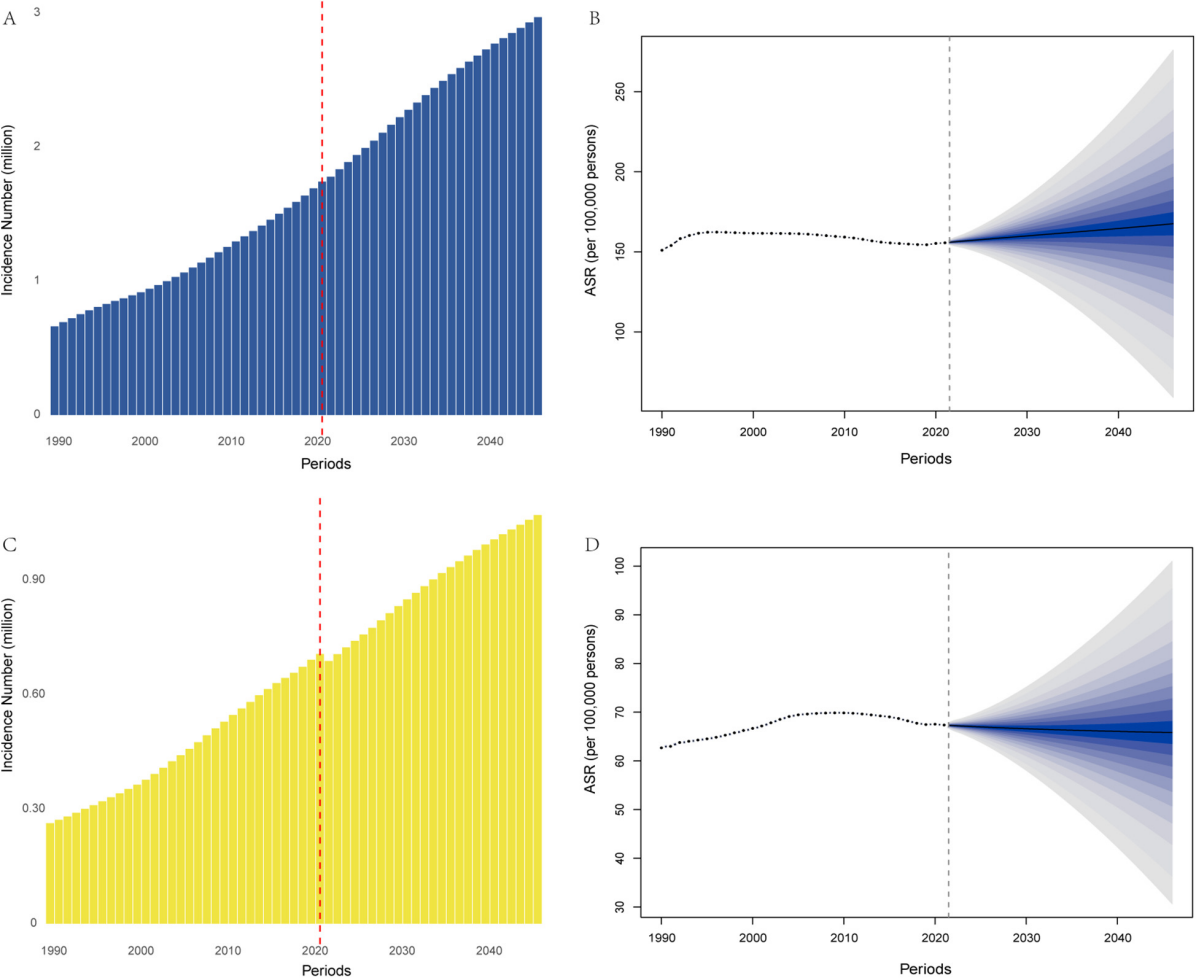
Trends in incidence number [**(A)**: females, **(C)**: males], and age-standardized incidence rate [**(B)**: females, **(D)**: males] in China from 1990 to 2021 and the predicted results from 2022 to 2046 by the BAPC model.

Using the BAPC model, we projected the PAD burden from 2021 to 2046 (Table 3). The estimated incidence number of PAD patients is projected to increase from 2.45 million in 2021 to 4.04 million by 2046, reflecting its rapid growth. Specifically, the number of female patients is expected to rise from 1.74 million in 2021 to 2.97 million in 2046 (Figure 3A), while the number of male patients will increase from 0.71 million in 2021 to 1.07 million (Figure 3C). However, the age-standardized incidence rate shows only a slight increase in females (Figure 3B), with a marginal declining trend in males (Figure 3D). Additionally, the trends in the number of DALYs, along with ASRs, exhibit similarities to those of incidence over the next 25 years. Further details are provided in Table 3 and Supplementary Figure S1.

**Table 3:** Prediction of the number (incidence: million; DALYs: thousand) and age-standardized rate (per 100,000 persons) of PAD disease burden stratified by gender in China over next 25 years.

Disease burden	Both				Female				Male			
	2021	2031	2041	2046	2021	2031	2041	2046	2021	2031	2041	2046
Incidence												
Number	2.45	3.13	3.78	4.04	1.74	2.28	2.77	2.97	0.71	0.85	1.01	1.07
ASR	112.63	115.83	119.85	123.53	155.63	160.40	165.04	167.54	67.35	66.54	66.16	65.83
DALYs												
Number	171.69	246.98	341.75	388.33	114.67	172.33	239.26	270.54	57.01	74.65	102.49	117.80
ASR	8.31	8.52	8.73	9.02	10.31	10.58	10.56	10.52	6.00	5.82	5.79	5.86

PAD, lower extremity peripheral arterial disease; ASR, age-standardized rate; DALYs, disability-adjusted life years.

## 4 Discussion

This study highlighted the PAD incidence and DALYs in 2021, historical trends from 1990 to 2021, and projections for the next 25 years. The disease burden in 2021 was more than twice in 1990 in terms of numbers. However, ASRs showed minimal changes and even a slight downward trend. This indicates that the increase in the absolute numbers was largely attributable to demographic changes associated with aging. This pattern is expected to continue, with the burden projected to double by 2046. Specifically, the incidence number is expected to reach 4.04 million new cases and corresponding to a ASIR of 123.53 per 100, 000 persons. Furthermore, the trends in the number and rate of DALYs exhibit a similarity to those of incidence. Consequently, given the anticipated changes over the next 25 years, it is imperative to place significant emphasis on PAD. Previous research has indicated that modifiable risk factors account for approximately 70% of the disease burden of PAD, highlighting the extent to which public health interventions can mitigate this burden by addressing factors such as smoking and physical activity (6). The increasing prevalence of risk factors for metabolic diseases, such as hypertension and diabetes, in China has also exacerbated the disease burden of PAD, underscoring the need for effective control measures (12). Prevention and management of lower limb atherosclerosis can significantly reduce the incidence and mortality of cardiovascular and cerebrovascular events, such as stroke and myocardial infarction. Thus, the findings of this study emphasize the severity of the disease burden posed by PAD and the necessity for its management. Additionally, this will provide a basis for the allocation of medical resources and the formulation of relevant policies. The ongoing establishment of PVMC across the country may help alleviate the growing burden of PAD to some extent, as these PVMCs consider every vascular segment.

We also conducted a separate analysis of the disease burden of PAD by sex and age. The burden of PAD was significantly higher in females than in males, which may be attributed to factors, such as lower pain threshold, higher prevalence of leg symptoms, and a greater likelihood of seeking medical care among females compared to males (24, 25). Additionally, the influence of sex hormones on cardiovascular diseases and atherosclerosis may be another contributing factor (26). In addition, previous research has indicated that the ankle-brachial index (ABI) used for diagnosing PAD tends to decrease with a reduction in height, with females exhibiting a lower height (27). However, it is important to note that over the past 30 years, the age-adjusted and age-specific disease burden of PAD among females has declined, while that among males has increased. This trend may be related to the higher prevalence of risk factors in men, including unhealthy lifestyle behaviors such as smoking and a greater incidence of metabolic diseases like hypertension and diabetes. Previous studies have indicated that smoking doubles the risk of PAD compared to non-smokers (28). Similarly, metabolic diseases such as diabetes significantly elevate the risk of developing asymptomatic or symptomatic PAD, with the incidence of intermittent claudication among diabetic individuals being 2 to 3 times higher than that of non-diabetic counterparts (29, 30). Although the burden of PAD among females remains significantly higher than among males, this pattern is likely to persist in the long term. Therefore, priority should be given to the prevention and management of PAD in women while also closely monitoring the changing burden of PAD among men.

At the same time, we examined the effects of period and cohort on the incidence and DALYs rates of PAD. The period relative risk (RR) showed an upward trend, likely due to increase in metabolic diseases, such as hypertension, diabetes, and dyslipidemia, along with unhealthy lifestyle behaviors like smoking (31). In contrast, the cohort RR demonstrated a downward trend, which may be attributed to improvements in healthcare standards and greater health awareness (32).

## 5 Limitations

The primary limitation of this study is the potential underestimation of the disease burden associated with PAD. First, some individuals‒particularly in resource-limited western regions‒ may not have sought medical care. Second, we may not have fully captured the burden of acute limb ischemia and chronic limb-threatening ischemia (6, 33). Furthermore, patients who have undergone amputation or who suffer from severe limb ischemia are often excluded from studies (34). Third, there are inconsistencies and inaccuracies in self-reported claudication data across regions. Additionally, the use of the ABI in diagnosing PAD remains controversial. Although ABI is a simple and non-invasive clinical test that has been used in practice for some time, there is a lack of standardization in measurement techniques and in defining abnormal cutoff values (34, 35). An ABI < 0.9 is estimated to have a sensitivity of less than 80% for detecting PAD (36).

## 6 Conclusion

Due to China's large population, aging demographic, and the rising prevalence of unhealthy lifestyles, the disease burden of PAD in China is substantial. The burden increased markedly from 1990 to 2021 and is expected to continue rising over the next 25 years. Greater efforts are needed to control modifiable risk factors‒such as smoking and metabolic diseases‒and to prevent and manage PAD through measures like the establishment of PVMC, thereby reducing the overall disease burden.

## Data Availability

Publicly available datasets were analyzed in this study. This data can be found here: https://vizhub.healthdata.org/gbd-results/.
