# Supplementary material for: Epidemic of lower extremity peripheral arterial disease in China: current trends and future prediction
Source: Front Cardiovasc Med. 2025 Jun 11;12:1571146. doi: 10.3389/fcvm.2025.1571146 (PMC12187828; doi:10.3389/fcvm.2025.1571146)
Supplement: Supplementary file 1 [file Table1.docx]

Table S1 The sex-age-specifc DALYs number and rate of PAD in 2021 and their percentage changes from 1990 to 2021

| **Categories** | **Female** | |  | **Male** | |
| --- | --- | --- | --- | --- | --- |
|  | **2021** | **AAPC, 95% CI** |  | **2021** | **AAPC, 95% CI** |
| **Number** |  |  |  |  |  |
| 40-44 years | 321 (229-430) | -0.14 (-0.95-0.67)* |  | 561 (380-783) | 1.45 (0.70-2.20)* |
| 45-49 years | 493 (352-664) | 1.51 (0.85-2.17)* |  | 863 (584-1216) | 3.51 (2.77-4.25)* |
| 50-54 years | 2105 (1278-3490) | 2.42 (1.99-2.85)* |  | 2044 (1455-2902) | 3.13 (2.78-3.48)* |
| 55-59 years | 6926 (3622-12742) | 2.32 (1.96-2.68)* |  | 4461 (2813-7268) | 2.83 (2.44-3.23)* |
| 60-64 years | 10202 (5284-19008) | 2.34 (2.04-2.65)* |  | 5783 (3651-9670) | 2.80 (2.45-3.16)* |
| 65-69 years | 20590 (9843-42028) | 2.43 (2.08-2.77)* |  | 10822 (6516-19073) | 3.00 (2.76-3.23)* |
| 70-74 years | 23497 (11527-46339) | 2.26 (2.05-2.48)* |  | 11419 (6996-19421) | 3.17 (3.01-3.34)* |
| 75-79 years | 20716 (10146-37552) | 2.75 (2.67-2.84)* |  | 9149 (5571-14067) | 4.11 (3.87-4.34)* |
| 80-84 years | 15881 (8269-28658) | 3.80 (3.71-3.89)* |  | 6595 (4429-9667) | 5.45 (5.28-5.61)* |
| 85+ years | 14018 (7795-23443) | 5.68 (5.56-5.79)* |  | 5313 (3861-7030) | 7.37 (7.22-7.53)* |
| **Rate** |  |  |  |  |  |
| 40-44 years | 0.72 (0.51-0.96) | -1.69 (-2.02--1.36)* |  | 1.20 (0.81-1.67) | -0.22 (-0.48-0.04) |
| 45-49 years | 0.91 (0.65-1.22) | -1.31 (-1.58--1.03)* |  | 1.54 (1.04-2.17) | 0.83 (0.45-1.20)* |
| 50-54 years | 3.53 (2.14-5.84) | -1.10 (-1.28--0.93)* |  | 3.34 (2.38-4.75) | -0.03 (-0.11-0.05) |
| 55-59 years | 12.58 (6.58-23.14) | -0.91 (-1.04--0.77)* |  | 8.13 (5.13-13.24) | -0.08 (-0.16-0.00)* |
| 60-64 years | 28.04 (14.53-52.25) | -0.76 (-0.87--0.66)* |  | 15.79 (9.97-26.40) | -0.05 (-0.14-0.03) |
| 65-69 years | 52.84 (25.26-107.86) | -0.68 (-0.76--0.59)* |  | 28.67 (17.27-50.54) | -0.08 (-0.16--0.01)* |
| 70-74 years | 85.63 (42.01-168.87) | -0.58 (-0.65--0.50)* |  | 44.16 (27.06-75.11) | -0.02 (-0.09-0.06) |
| 75-79 years | 118.27 (57.92-214.38) | -0.47 (-0.54--0.39)* |  | 58.64 (35.70-90.16) | 0.19 (0.09-0.29)* |
| 80-84 years | 142.86 (74.38-257.79) | -0.38 (-0.45--0.31)* |  | 76.02 (51.06-111.43) | 0.48 (0.35-0.61)* |
| 85+ years | 161.38 (89.74-269.89) | -0.28 (-0.37--0.20)* |  | 120.46 (87.54-159.39) | 0.90 (0.69-1.12)* |
