# Supplementary figures and images for: Epidemic of lower extremity peripheral arterial disease in China: current trends and future prediction
Source: Front Cardiovasc Med. 2025 Jun 11;12:1571146. doi: 10.3389/fcvm.2025.1571146 (PMC12187828; doi:10.3389/fcvm.2025.1571146)

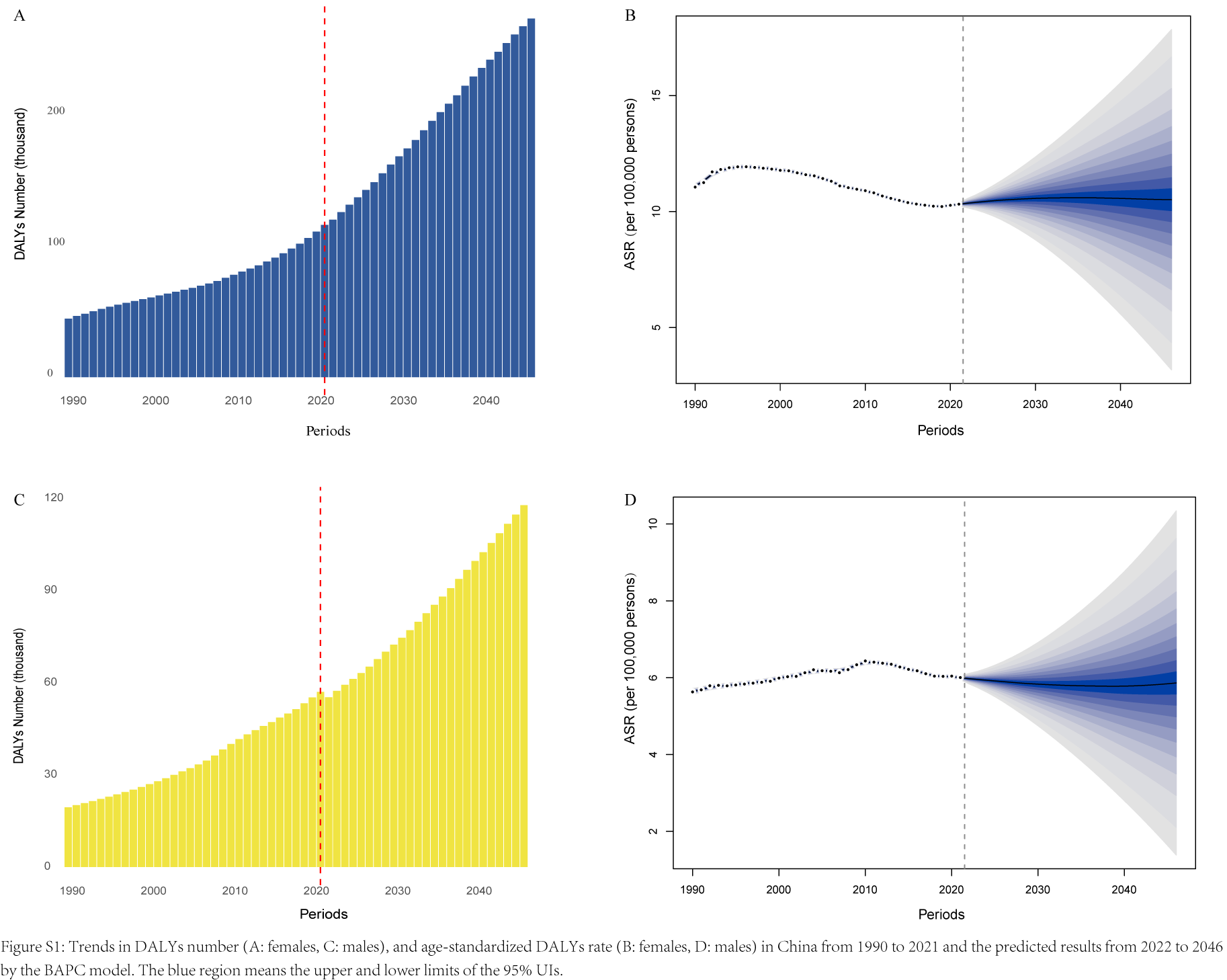

Supplement: Supplementary file 2 [file Image1.tif]
